# Kinematic mechanism of the rehabilitative effect of 4-channel NMES: post-hoc analysis of a prospective randomized controlled study

**DOI:** 10.1038/s41598-023-40359-3

**Published:** 2023-08-18

**Authors:** Jiwoon Lim, Jun Chang Lee, Eun Gyeong Jang, Sun Young Choi, Kyoung-Ho Seo, So Young Lee, Donghwi Park, Byung-Mo Oh, Han Gil Seo, Ju Seok Ryu

**Affiliations:** 1 Department of Rehabilitation Medicine, Korea University Ansan Hospital, Ansan-si, South Korea; 2 Department of Rehabilitation Medicine, Seoul National University Bundang Hospital, Seongnam-si, South Korea; 3 Department of Rehabilitation Medicine, Seongnam Citizen’s Medical Center, Seongnam-si, South Korea; 4 Department of Rehabilitation Medicine, Jeju National University Hospital, Jeju National University College of Medicine, Jeju, South Korea; 5 Department of Rehabilitation Medicine, Ulsan University Hospital, Ulsan, South Korea; 6 Department of Rehabilitation Medicine, Seoul National University College of Medicine, Seoul National University Hospital, Seoul, South Korea; 7 Department of Rehabilitation Medicine, Seoul National University Bundang Hospital, Seoul National University College of Medicine, 82 Gumi-ro 173 Beon-gil, Bundang-gu, Seongnam-si, 463-707 Gyeonggi-do South Korea

**Keywords:** Diseases, Pathogenesis

## Abstract

The sequential 4-channel neuromuscular electrical stimulation (NMES), based on the normal contractile sequences of swallowing-related muscles, is a new rehabilitative treatment. The objective of this study was to explore the mechanism of the rehabilitative effect of the 4-channel NMES using kinematic analysis of videofluoroscopic swallowing study (VFSS) data. For this post-hoc analysis, we included a subset of participants from the prospective randomized controlled study on the clinical effectiveness of the sequential 4-channel NMES compared with that of the conventional 2-channel NMES. Seventeen subjects (11 and six in the 4- and 2-channel NMES groups, respectively) were eligible for the kinematic analysis of VFSS data. The hyoid bone movement was analyzed by evaluating the distance and time parameters with four peak points (A, B, C, D). The 4-channel NMES group showed significant improvement in vertical distances (A–C), horizontal distance (A–B, A–C), time interval (A–B–C) and total time, compared with their pretreatment data. The 2-channel NMES group showed significant improvements in time interval (A–B); however, the Euclidean distance (A–D) and mean velocity of the Euclidean distance (A-C) were significantly decreased. When the two groups were directly compared, the 4-channel group showed significantly greater improvement in horizontal distance (A–B), Euclidean distance (A–D), time interval (A–B–C), and mean velocity the Euclidean distance (A–D). The results in this study suggest that the sequential 4-channel NMES might lead to the physiologic circular movement of the hyoid bone during swallowing, and therefore be an effective treatment for dysphagia.

Trial registration: Clinicaltrials.gov, registration number: NCT03670498.

## Introduction

Dysphagia is one of the most common and serious problems in patients with stroke and its prevalence ranges from 37 to 78%^1^. Decreased laryngeal elevation due to pharyngeal muscle weakness is the main cause of dysphagia in patients with stroke, which can induce aspiration and pharyngeal residue during swallowing^2^. There are several methods for treating dysphagia, including oropharyngeal exercise, compensatory maneuvers, and neuromuscular electrical stimulation (NMES)^3^.

The 2-channel NMES, one of the most common therapeutic methods for dysphagia, is known to strengthen swallowing-related muscles by motor stimulation and facilitating the swallowing reflex through sensory stimulation^4^. However, the precise mechanism of the 2-channel NMES treatment is yet to be determined, and there are controversies regarding its efficacy and the method of stimulation^5^. Current evidence is insufficient to indicate that NMES is superior to swallowing therapy, and a recent randomized controlled trial failed to prove the effectiveness of the 2-channel NMES in patients with subacute stroke^4,6^.

Our previous study demonstrated that the suprahyoid muscles are activated approximately 150-350 ms earlier than the infrahyoid muscles. These sequential contractions of the swallowing-related muscles cause a circular movement of the hyoid bone during normal swallowing^7,8^. These results suggest that co-stimulation of the suprahyoid and infrahyoid muscles via the conventional 2-channel NMES could result in the cancellation of positive effects^9,10^. However, unlike the 2-channel NMES, the sequential 4-channel NMES was recently developed and its action is based on the normal physiological contractile sequence of swallowing-related muscles. The stimulation of these muscles via the 4-channel NMES may lead to better adjustment of the abnormal hyoid and laryngeal motion in patients with dysphagia^11^.

In our previous structured studies, the sequential 4-channel NMES showed significant compensatory effects in clinical and kinematic parameters during swallowing^12^. Moreover, in a recent randomized controlled study comparing rehabilitative effects of the sequential 4-channel NMES to the 2-channel NMES, the 4-channel NMES showed significant clinical improvement in penetration-aspiration scale and videofluoroscopic dysphagia scale scores. These results suggest that the sequential 4-channel NMES, through its activation of the suprahyoid, thyrohyoid, and other infrahyoid muscles mimics physiological activation patterns and may therefore be a new effective treatment for dysphagia^13^.

However, the mechanism by which clinical improvement in swallowing occurs when 4-channel NMES is used has not yet been fully elucidated. The videofluoroscopic swallowing study (VFSS) is considered the gold standard evaluation of dysphagia, and a kinematic analysis of VFSS data can provide quantitative information about the hyoid movement and bolus, detecting subtle abnormalities of swallowing^14,15^. However, the manual kinematic analysis is labor intensive, time consuming, and the accuracy of the result can vary depending on the skill of the examiner. In order to overcome these problems, we developed and validated an automated kinematic analysis program (AKAP) that analyzed the two-dimensional moving trajectory of the hyoid bone via a visual tracking method^16^. In a previous study, it was useful for determining the pathophysiology of dysphagia^17^.

Therefore, a clearer understanding of how the sequential 4-channel NMES shows the rehabilitative effectiveness is needed. The aim of this study was to investigate the mechanism of the rehabilitative effects of the sequential 4-channel NMES using kinematic analysis of the hyoid bone movement, using data from a randomized controlled trial^13^. We hypothesized that the vertical and horizontal movement extents, as well as the duration of hyoid movements in the 4-channel NMES may be different from those in the 2-channel NMES.

## Methods

### Study design and participants

This was a post-hoc analysis of a multicenter, prospective, double-blind, randomized controlled clinical trial on the clinical effectiveness of the sequential 4-channel NMES compared with that of the conventional 2-channel NMES for the treatment of dysphagia. The detailed trial design and study results have been published previously^13^. Briefly, from October 2018 to August 2019, patients from five hospitals were identified who met the following criteria; (1) within 3 months of a cerebral infarction or hemorrhage diagnosis, (2) at least one symptom of dysphagia, and (3) confirmed diagnosis of dysphagia via VFSS. The exclusion criteria were: (1) severe cognitive dysfunction and could not follow 1-step commands; (2) serious psychiatric disorder; and (3) a history of cervical surgery and respiratory difficulty. Patients who were pregnant or breastfeeding, patients who had cancer, and patients who had allergic reactions to the electrodes of NMES were also excluded.

**Figure 1:**
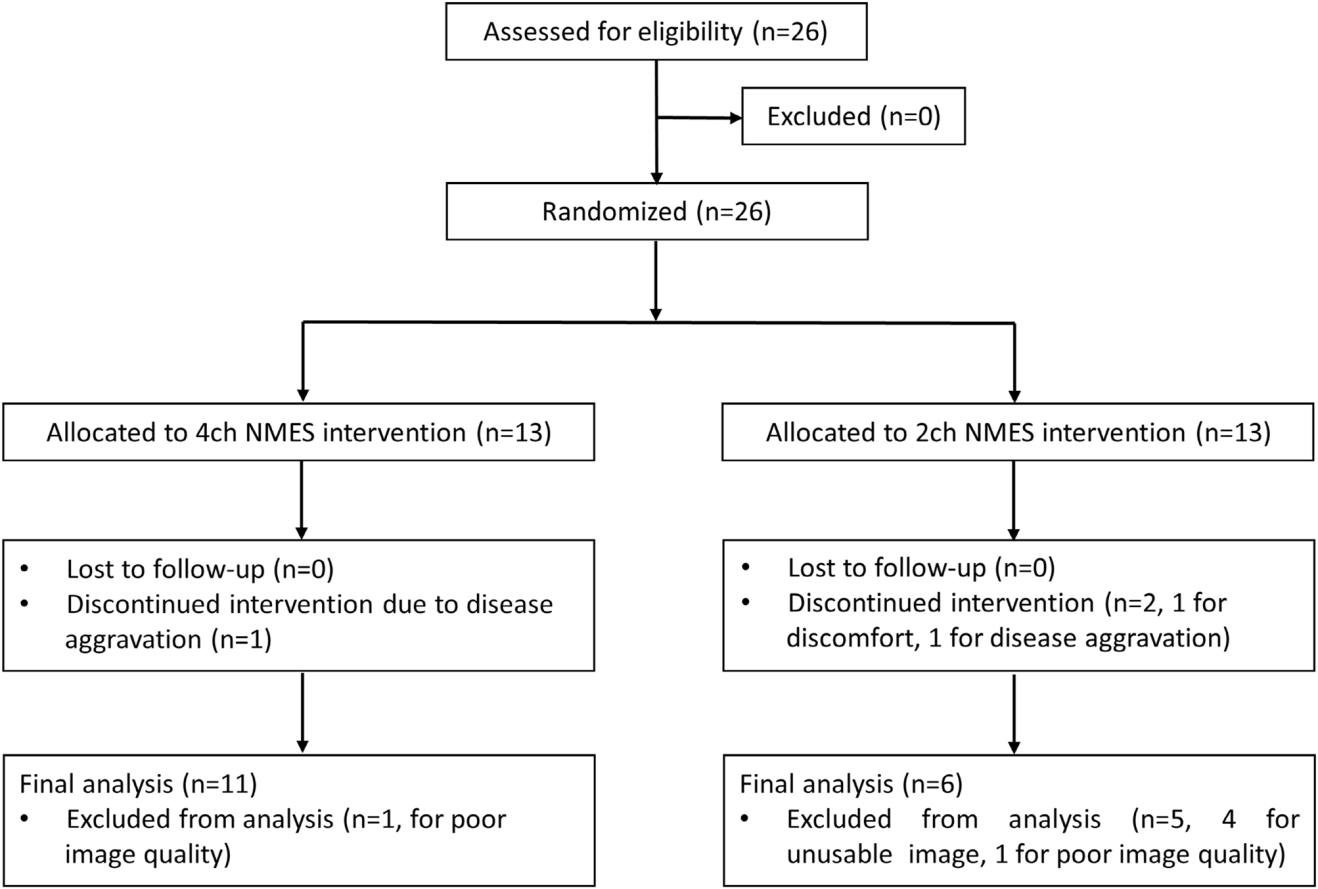
Flowchart of this study.

For this post-hoc analysis, we included a subset of participants who’s initial and follow-up VFSS data could be kinematically identified and analyzed (Fig. 1). Exclusion criteria for analyzing hyoid bone tracking of VFSS data included low-image resolution, inappropriate photography conditions, and too much head/neck shaking during swallowing. These factors led to excessive missing frames during the automatic hyoid bone tracking process, and therefore, these cases were excluded from the current study.

Patients were assigned in a 1:1 ratio to receive the 4-channel NMES or 2-channel NMES. All participants received 2- or 4-channel NMES for 2–3 weeks (minimal session: 7 times, treatment duration: 300–800 min). The interventions started within 1 week after the initial clinical and VFSS evaluations, and follow-up evaluations were performed within 1 week after the last intervention. The maximal duration between the initial and follow-up evaluations was 4 weeks. The reporting of this study conforms to all CONSORT 2010 guidelines. The study protocol was approved by the institutional review board of each hospital (IRB Nos.: E-1806/475-002, 2018-07-012, JEJUNUH 2018-07-010, J-1810-064-979, DFH19DPOS033, respectively), approved by the Ministry of Food and Drug Safety in the Republic of Korea, and registered at clinicaltrial.gov (registration number: NCT03670498, initial release: 13/09/2018, first registration date: 01/10/2018, actual study completion date: 04/08/2019, last release: 23/07/2020). Written informed consent was obtained from all participants.

### Equipment: sequential 4-channel NMES and 2-channel NMES

The sequential 4-channel NMES has four channels with adaptable amplitude, frequency, duration, and latency of current. (Supplementary Fig. S1a: STF-1000, Stratec Co., Ltd, Anyang, South Korea). It also includes four pairs of round-shaped electrodes which are 22 mm in diameter. The gaps between the electrodes were either 0.5 cm (type 1 electrode) or 1 cm (type 2 electrode). The type 1 electrode was used for channels 1, 2, and 4, and the type 2 electrode was used for channel 3 (Supplementary Fig. S1b; One Bio Medic Co., Ltd, Bucheon-si, Gyeonggi-do, South Korea).

Channel 1 (right) and channel 2 (left) electrodes were placed superior to the hyoid bone and posterior to the mandible, 1 cm lateral to the midline, and the targeted muscles were the bilateral digastric and mylohyoid muscles. Channel 3 electrodes were placed on the bilateral superior poles of the thyroid cartilage to target the bilateral thyrohyoid muscles, and channel 4 electrodes were placed medial to the sternocleidomastoid muscle and inferior to the thyroid cartilage, and the targeted muscles were the other infrahyoid muscles (sternohyoid, omohyoid, and sternothyroid muscles) (Supplementary Fig. S2a). All electrical stimulation parameters were based on previous studies^7,11^. Electrical stimulation was started in channels 1 and 2 first, and stimulation via channels 3 and 4 started 150 ms and 250 ms later, respectively. The stimulation durations of channels 1, 2, 3, and 4 were 1200 ms, 1200 ms, 1050 ms, and 950 ms respectively. Hence, all stimulations in the sequence ended concurrently^7^.

In the 2-channel NMES group, electrical stimulation was applied via two sets of electrodes attached to the suprahyoid and thyrohyoid muscles, whereas no electrical stimulation was applied to the other electrodes that were placed for blinding purposes (Supplementary Fig. S2b). The 2-channel NMES (Vitalstim^®^; Chattanooga Group, Hixson, TN, USA) and 4-channel NMES had the same stimulation parameters. The pulse frequency, duration, and interphase interval were 80 Hz, 300 µs, and 100 µs, respectively. The stimulus intensity was increased until the subjects could no longer tolerate the discomfort or pain (between 0 and 25 mA)^18^.

### Kinematic analysis of videofluoroscopic swallowing study data

For the VFSS, subjects were tested with the following boluses sequentially: thin fluid (International dysphagia diet standardization initiative (IDDSI) 0), mildly thick fluid (IDDSI 2), soft and bite-sized food (IDDSI 6), and regular food (IDDSI 7)^19^. Each patient received an initial 3 mL bolus, followed by two 5 mL boluses. Fluids (thick and thin) were delivered using 10-mL syringes and the others were consumed using a spoon. All fluoroscopic images taken during swallowing were digitally recorded.

**Figure 2:**
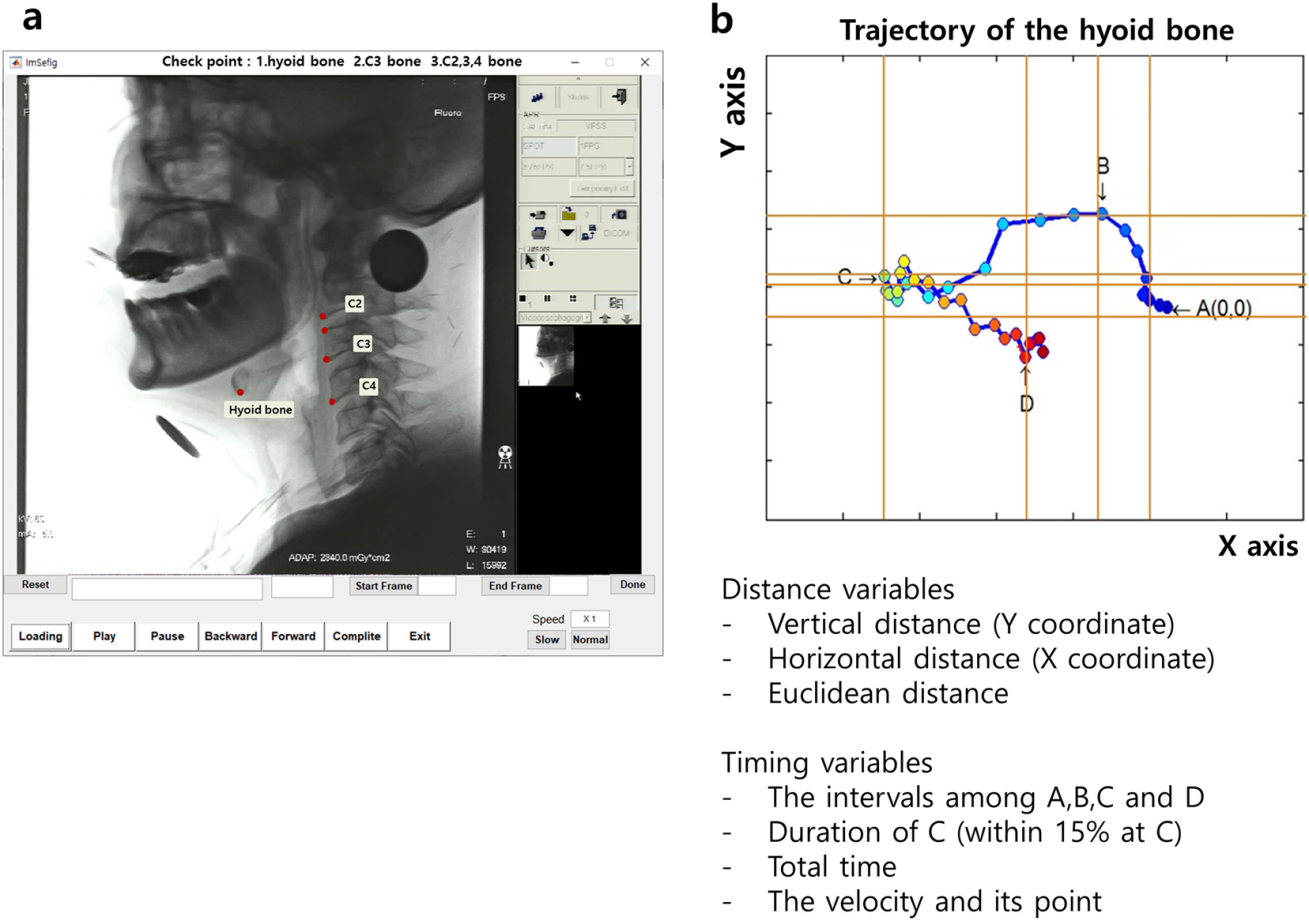
This figure shows the automated kinematic analysis program (AKAP, C-2015-019815). (**a**) After marking the anterior border of the hyoid bone, the anterosuperior and anteroinferior edges of the C3 vertebral body and anteroinferior edges of the C2, C3, and C4 vertebral bodies are marked in the first frame of the recording. (**b**) Then, the MATLAB program automatically analyzed the position of the hyoid bone based on the position of C2, C3, and C4 vertebral bodies from the first to the last frame of the recording. AKAP measures the distance and timing parameters between the points (A–D). A(0, 0): the starting point of the hyoid bone when swallowing, B: the most upward point, C: the most anterior location, D: the endpoint of swallowing.

Kinematic analysis was performed for the VFSS video clips. VFSS video clips were cut and recorded at 30 images/s. Two-dimensional hyoid bone tracking was performed using AKAP based on MATLAB (The MathWorks Inc., Natick, MA)^16^. In the first image, we marked the reference points in the following manner: the anterior border of the hyoid bone, anterosuperior and anteroinferior edge of the C3 vertebral body, and anteroinferior edge of the C2, C3, and C4 vertebral bodies (Fig. 2a). Afterwards, AKAP automatically marked the remaining frames and analyzed hyoid bone movement based on the reference points (Fig. 2b).

Based on the two-dimensional moving trajectory of the hyoid bone, four points were identified: point A was the starting point of the hyoid bone when swallowing, point B was the most upward point, point C was the most anterior location (equilibrium state of supra-and infrahyoid muscles), and point D was the endpoint of swallowing (Fig. 2b). We calculated the distance and timing parameters for each point from A to D. The AKAP variables include the vertical distance between A–B, A–C, B–C, and A–D; the horizontal distance between A–B, A–C, and A–D; the Euclidean distance between A–D; the time interval between A–B, between A–B–C, between A–B–C–D; total time; and mean velocity of the Euclidean distances between A–B, A–C, and A–D (Fig. 2(b))^16^.

### Statistical analysis

As this was a pilot study, a formal sample size calculation was not conducted. The main aim of this pilot study was to determine feasibility of the 4-channel NMES for the main study and to estimate the effect size to inform a future sample size calculation for the main clinical trial. Therefore, a sample of 24 participants was chosen (12 in each group). Considering a dropout rate of 10%, the final sample size was determined to be a total of 26 participants^20^.

Descriptive statistics were used to summarize participant characteristics using mean (SD) or number (percentage) as appropriate. Continuous data were assessed for skewness by visual inspection and using a normality test. The Mann − Whitney test was used to compare the two groups. The paired t-test or Wilcoxon signed rank test (if the assumptions of parametric statistical analysis were not satisfied) was used to compare data from the initial and follow-up evaluations. The Statistical Package for the Social Sciences software (version 25.0; SPSS Inc., Chicago, IL, USA) was used for all statistical analyses. Statistical significance was set at P < 0.05.

### Ethics approval and consent to participate

The study protocol was approved by the institutional review board of each hospital (IRB No.: E-1806/475-002, 2018-07-012, JEJUNUH 2018-07-010, J-1810-064-979, DFH19DPOS033) and all methods were performed in accordance with relevant guidelines and regulations. All patients or their representatives provided written informed consent prior to study participation. The study was also approved by the Ministry of Food and Drug Safety in Republic of Korea.

## Results

Seventeen participants (11 and six in the 4- and 2-channel NMES groups, respectively) were eligible for the kinematic analysis of VFSS data. A total of 198 VFSS video clips (121 and 77 in the 4- and 2-channel NMES groups, respectively) were analyzed. The demographic data of the participants are presented in Table 1. The average ages of the 4- and 2-channel NMES groups were 67.7 ± 13.9 and 59.3 ± 15.3 years, respectively. The treatment durations of the 4- and 2-channel groups were 413.6 ± 69.0 and 405.0 ± 56.1 min, respectively. There were no significant differences in the demographic data between the two groups.

**Table 1:** Demographic data of patients.

	4ch NMES (n = 11)	2ch NMES (n = 6)	P-value
Age, years	67.7 (± 13.9)	59.3 (± 15.3)	0.22
Sex	Male 6 (54.5%) Female 5 (45.4%)	Male 4 (66.7%) Female 2 (33.3%)	1.00
Hypertension	2 (18.2%)	3 (50.0%)	0.28
DM	0 (0%)	0 (0%)	–
Prior CVA	8 (72.7%)	5 (83.3%)	1.00
Smoking Hx	0 (0%)	2 (33.3%)	0.11
Cervical op	0 (0%)	0 (0%)	–
MMSE-K	16.1 (± 10.5)	13.3 (± 7.9)	0.88
NMES treatment duration, min	413.6 (± 69.0)	405.0 (± 56.1)	0.88
Stroke: ICH	8:3	3:3	
Stroke territory			
ACA territory	0 (0.0%)	0 (0.0%)	
MCA territory	4 (50.0%)	2 (66.7%)	
PCA territory	0 (0.0%)	0 (0.0%)	
Brainstem lesion	4 (50.0%)	1 (33.3%)	
VFSS images	121	77	

Continuous variables are represented as means (± SD), Categorical variables are represented N and/or (%).

*DM* diabetes mellitus, *CVA* cerebrovascular accident, *NMES* neuromuscular electrical stimulation, *ACA* anterior cerebral artery, *MCA* middle cerebral artery, *PCA* posterior cerebral artery.

In a comparison between pre- and post-treatment evaluations in the 4-channel NMES group, the horizontal distance of A–B was significantly increased, and the time interval between A–B–C and total time were significantly reduced during thin fluid (IDDSI 0) swallowing. Additionally, the vertical distance of A–C, the horizontal distance of A–B, and the horizontal distance of A–C were significantly increased during soft and regular food intake (IDDSI 6, 7) (Tables 2, 3).

**Table 2:** The comparisons of spatial parameters between before and after treatment in both groups.

Spatial parameters (pixel)	4ch NMES			2ch NMES		
	Pre	Post	p-value	Pre	Post	p-value
IDDSI 0						
Vertical A–B	26.7 ± 20.9	24.4 ± 17.1	0.63	34.8 ± 18.5	16.4 ± 11.2	0.13
Vertical A–C	13.0 ± 24.5	20.0 ± 16.5	0.31	26.3 ± 14.1	14.5 ± 12.6	0.24
Horizontal A–B	**5.4 ± 9.0**	**19.4 ± 23.8**	0.03*	20.0 ± 13.1	10.3 ± 9.8	0.18
Horizontal A–C	23.1 ± 14.2	26.2 ± 22.8	0.51	25.6 ± 15.7	13.0 ± 9.1	0.13
Euclidean A–D	134.1 ± 46.8	134.1 ± 50.9	1.00	111.6 ± 56.8	80.5 ± 29.9	0.18
IDDSI 2						
Vertical A–B	24.1 ± 18.4	28.8 ± 22.1	0.14	19.6 ± 14.1	11.0 ± 9.6	0.50
Vertical A–C	15.2 ± 22.0	20.8 ± 25.0	0.26	7.2 ± 16.3	5.3 ± 7.5	0.69
Horizontal A–B	17.7 ± 28.0	25.8 ± 33.2	0.21	8.8 ± 10.8	6.3 ± 9.5	0.23
Horizontal A–C	32.7 ± 23.8	34.6 ± 30.9	0.86	22.7 ± 12.5	14.7 ± 7.7	0.35
Euclidean A–D	141.1 ± 54.7	170.2 ± 153.1	0.77	124.7 ± 53.6	83.7 ± 45.6	0.35
IDDSI 6, 7						
Vertical A–B	21.0 ± 12.0	23.6 ± 15.5	0.347	22.2 ± 21.7	22.0 ± 12.2	0.89
Vertical A–C	**11.4 ± 15.6**	**19.9 ± 16.7**	0.03*	19.7 ± 23.1	18.2 ± 10.9	0.89
Horizontal A–B	**6.4 ± 11.0**	**18.5 ± 16.2**	0.03*	17.2 ± 22.5	15.9 ± 16.5	0.69
Horizontal A–C	**24.4 ± 18.2**	**31.4 ± 18.1**	0.045*	23.7 ± 24.7	24.2 ± 12.9	0.50
Euclidean A–D	123.7 ± 32.9	138.6 ± 56.5	0.34	**155.6 ± 49.2**	**127.1 ± 53.8**	0.04*

Values represent means ± SD, *P < 0.05.

Significant values are in bold.

**Table 3:** The comparisons of temporal parameters between before and after treatment in both groups.

Temporal parameters	4ch NMES			2ch NMES		
	Pre	Post	p-value	Pre	Post	p-value
IDDSI 0						
Time A–B, s	0.4 ± 0.3	0.3 ± 0.2	0.54	**0.5 ± 0.2**	**0.3 ± 0.1**	0.046*
Time A–B–C, s	**0.7 ± 0.5**	**0.4 ± 0.3**	0.03*	0.6 ± 0.3	0.3 ± 0.1	0.06
Total time, s	**1.5 ± 0.6**	**1.3 ± 0.5**	0.04*	0.9 ± 0.4	0.7 ± 0.2	0.35
Velocity† A–C, pixel/s	1.7 ± 0.8	1.6 ± 0.9	0.47	1.1 ± 0.6	0.9 ± 0.4	1.00
Velocity A–D, pixel/s	0.8 ± 0.5	1.0 ± 0.5	0.08	**0.2 ± 0.1**	**0.6 ± 0.3**	0.02*
IDDSI 2						
Time A–B, s	0.4 ± 0.2	0.4 ± 0.2	0.80	0.4 ± 0.1	0.2 ± 0.2	0.35
Time A–B–C, s	0.7 ± 0.4	0.6 ± 0.3	0.60	0.5 ± 0.1	0.4 ± 0.2	0.23
Total time, s	1.5 ± 0.3	1.4 ± 0.9	0.37	1.1 ± 0.3	0.7 ± 0.3	0.07
Velocity A–C, pixel/s	1.9 ± 0.9	2.0 ± 1.7	0.60	1.5 ± 0.8	1.0 ± 0.5	0.69
Velocity A–D, pixel/s	0.9 ± 0.6	0.9 ± 0.8	0.95	0.3 ± 0.2	0.3 ± 0.3	0.50
IDDSI 6,7						
Time A–B, s	0.4 ± 0.3	0.5 ± 0.5	0.39	1.0 ± 0.9	0.3 ± 0.2	0.23
Time A–B–C, s	0.5 ± 0.3	0.6 ± 0.6	0.58	1.2 ± 0.8	0.4 ± 0.2	0.14
Total time, s	1.4 ± 0.4	1.3 ± 0.7	0.73	1.6 ± 1.0	1.1 ± 0.8	0.28
Velocity A–C, pixel/s	1.8 ± 0.8	1.7 ± 0.8	0.41	**1.4 ± 0.3**	**1.3 ± 0.4**	0.04*
Velocity A–D, pixel/s	0.8 ± 0.4	0.7 ± 0.4	0.20	0.3 ± 0.2	0.6 ± 0.4	0.23

Values represent means ± SD, *P < 0.05.

^†^Velocity represents the mean velocity of the Euclidean distance between two points.

Significant values are in bold.

In the pre- and post-treatment comparisons within the 2-channel NMES group, the time interval between A–B was significantly reduced, and the mean velocity of the Euclidean distance A–D was significantly increased during thin fluid (IDDSI 0) swallowing. However, the Euclidean distance of A–D and the mean velocity of the Euclidean distance A–C were significantly decreased (Tables 2, 3).

**Figure 3:**
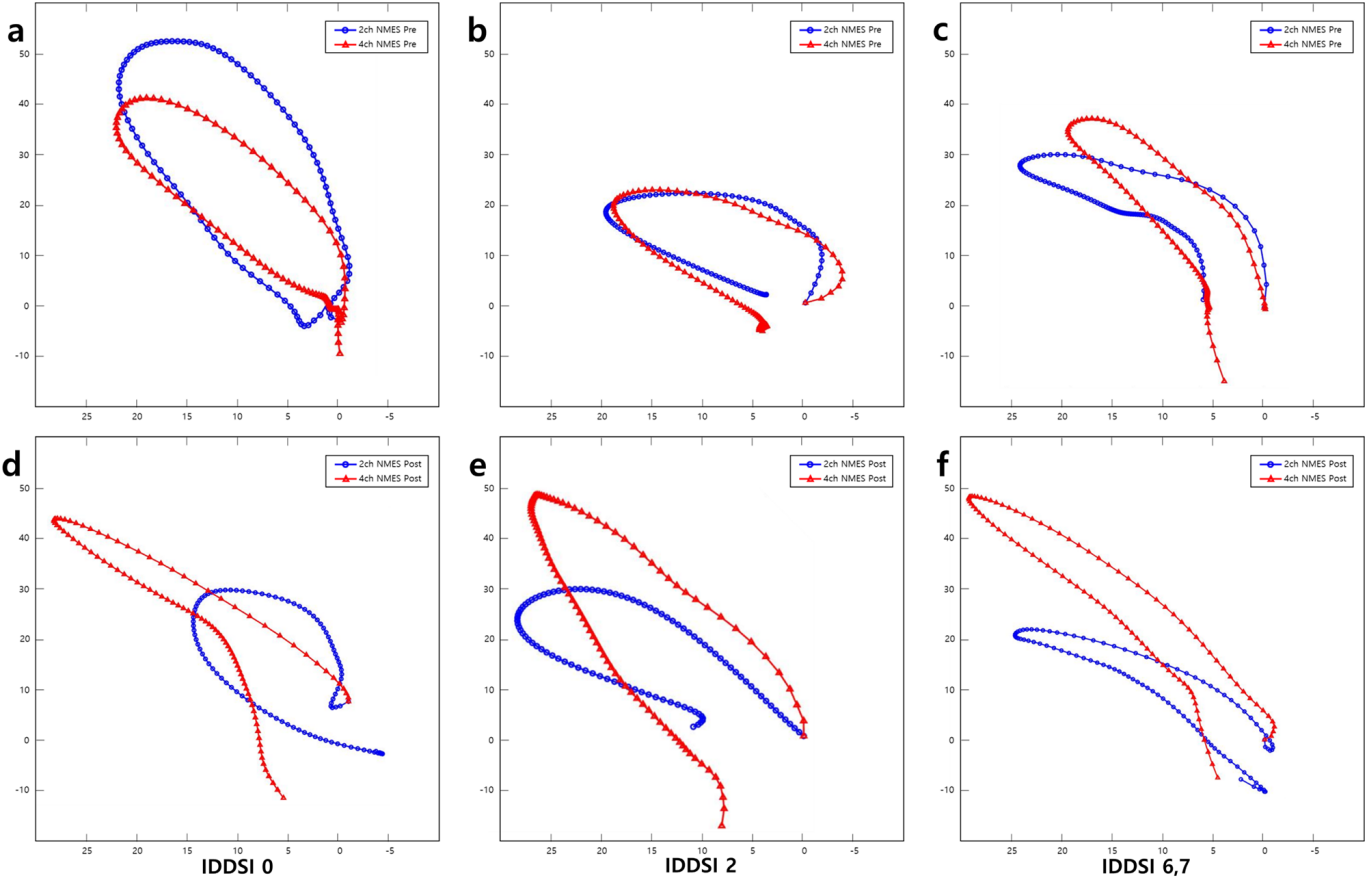
Comparison of the movement trajectory of hyoid bone before and after 2-channel NMES group (blue and circle line) and 4-channel NMES group (red and triangle line). Though the 2-channel NMES group produced hyolaryngeal descent in thin fluid (IDDSI 0) (**a,d**), mildly thick fluid (IDDSI 2) (**b,e**), and soft and regular food (IDDSI 6, 7) (**c,f**), the 4-channel NMES group showed increased the horizontal, vertical, and Euclidean distance parameters after the treatments.

Figure 3 shows the examples of 2D hyoid bone trajectory graphs between 2-channel NMES and 4-channel NMES. After the treatments, the movement trajectory of the hyoid bone became larger and clear in the superior-anterior direction with 4-channel NMES than 2-channel NMES.

Changes in the parameters between the two groups were compared. When swallowing thin fluid (IDDSI 0), the horizontal A-B distance was significantly longer in the 4-channel NMES group than in the 2-channel NMES group. When swallowing soft and regular food (IDDSI 6, 7), the Euclidean distance of A–D was significantly longer, and the mean velocity of the Euclidean distance A–D was significantly higher in the 4-channel NMES group. The duration of A–B–C was significantly longer in the 4-channel groups (Table 4).

**Table 4:** The comparison of changes in the evaluated parameters between groups.

	IDDSI 0			IDDSI 2			IDDSI 6,7		
	4ch NMES	2ch NMES	p-value	4ch NMES	2ch NMES	p-value	4ch NMES	2ch NMES	p-value
Spatial parameters, pixel									
Vertical A–B	−2.3 ± 18.8	−18.5 ± 26.8	0.31	4.7 ± 8.5	−8.5 ± 23.4	0.36	2.6 ± 9.0	−0.2 ± 17.8	0.96
Vertical A–C	7.1 ± 26.9	−11.7 ± 23.1	0.22	5.5 ± 10.6	−1.9 ± 17.1	0.61	8.5 ± 11.9	−1.5 ± 22.1	0.51
Horizontal A–B	**14.0 ± 23.2**	−**9.7 ± 15.9**	0.02*	8.1 ± 18.4	−2.5 ± 6.0	0.36	12.1 ± 17.3	−1.3 ± 32.8	0.57
Horizontal A–C	3.1 ± 17.9	−12.7 ± 20.5	0.14	1.9 ± 16.4	−8.0 ± 16.8	0.70	7.0 ± 10.8	0.4 ± 25.9	0.65
Euclidean A–D	0.0 ± 39.7	−31.1 ± 65.1	0.45	29.1 ± 119.2	−41.0 ± 86.2	0.61	**14.8 ± 51.2**	−**28.5 ± 15.8**	0.04*
Temporal parameters									
Time A–B, s	−0.1 ± 0.4	−0.2 ± 0.2	0.14	0.0 ± 0.2	−0.1 ± 0.3	0.36	0.1 ± 0.5	−0.7 ± 0.9	0.16
Time A–B–C, s	−0.3 ± 0.5	−0.3 ± 0.3	0.62	−0.1 ± 0.3	−0.1 ± 0.2	0.61	**0.1 ± 0.6**	−**0.8 ± 0.8**	0.048*
Total time, s	−0.2 ± 0.4	−0.1 ± 0.3	0.67	v0.2 ± 0.8	−0.3 ± 0.3	1.00	−0.1 ± 0.6	−0.6 ± 0.9	0.72
Velocity A–C, pixel /s	−0.1 ± 0.6	−0.2 ± 0.9	0.77	0.1 ± 1.4	−0.5 ± 1.3	1.00	−0.1 ± 0.4	−0.1 ± 0.1	0.88
Velocity A–D, pixel /s	0.2 ± 0.5	0.4 ± 0.2	0.25	0.1 ± 0.7	−0.1 ± 0.3	1.00	**-0.1 ± 0.3**	**0.3 ± 0.5**	0.048*

Values represent means ± SD, *P < 0.05.

Significant values are in bold.

## Discussion

The purpose of this study was to investigate the mechanism of the rehabilitative effects of the sequential 4-channel NMES compared with that of the conventional 2-channel NMES, through kinematic analysis of hyoid bone movement. In this study, we observed differences in the spatial and temporal parameters of hyoid bone movement in the 4- and 2-channel NMES groups. Only the sequential 4-channel NMES group showed significant increase in the horizontal distance of A–B and significant decrease in the total time when swallowing thin fluid. Furthermore, we found that the horizontal distance of A–B and A–C, and the vertical distance of A–C in soft and regular food swallowing were significantly increased. According to our previous study^11^, the sequential 4-channel NMES facilitated the movement of hyolaryngeal structures during swallowing, which was proven through the significant decrease in time intervals of A–B–C and A–B. In particular, in our study, when we directly compared the improvement between the two groups, the 4-channel group was superior to the 2-channel group in terms of the Euclidean distance and velocity (soft and regular food; Table 4). These findings indicate that the hyoid bone was drawn more and longer in the superior-anterior direction and experienced greater circular trajectory movements in the 4-channel NMES group.

In a previous study, 2-channel conventional NMES improved pharyngeal peristalsis and cricopharyngeal functions at the esophageal entry but did not cause elevation of the hyolaryngeal complex^21^. It was also shown that co-stimulation of the submental and throat regions in the 2-channel NMES produced hyolaryngeal descent, causing movement in the opposite direction from that required for swallowing. These results suggest that placing electrodes over the anterior neck region might activate the sternohyoid and omohyoid muscles, rather than the muscles that produce hyolaryngeal elevation, such as the thyrohyoid and suprahyoid muscles^22^. Previous studies have shown NMES to be effective in increasing the cross sectional area and the muscle strength of type II myofibers^23,24^. Therefore, this hyolaryngeal descent caused by the application of the 2-channel conventional NMES is not a physiologic motion, and the main mechanism of the 2-channel NMES is to improve swallowing-related muscle strength. These findings were similar to our results in which the Euclidean distance of A–D and the time interval between A–B–C (soft and regular food) were significantly decreased after 2 weeks of treatment with the 2-channel NMES (Table 4, P < 0.05). This suggests that not only is the hyoid bone movement delayed, but the overall circular movement is also decreased; therefore, the conventional 2-channel NMES could result in the cancellation of positive swallowing function^9,10^.

However, the stimulation algorithm of the sequential 4-channel NMES is based on a normal contractile sequence. In the electromyography analysis, the suprahyoid muscles are activated about 150 ms and 350 ms earlier that of thyrohyoid and other infrahyoid (sternohyoid, sternothyroid, and omohyoid) muscles^7^. These sequential contractions of the suprahyoid and infrahyoid muscles cause a circular motion of the hyoid bone, initially moving forward–upward and then moving backward–downward^25^. After 2 weeks of treatment with the 4-channel NMES, the Euclidean distance of A–D and time interval between A–B–C (soft and regular food) were significantly increased, indicating that hyoid bone movement was changed in the direction of restoring the normal swallowing pattern (Table 4, P value < 0.05). Therefore, the main mechanism of the sequential 4-channel NMES is not only strengthening the muscles but also improving the coordination of swallowing muscles.

The horizontal A–B distance (thin fluid) was significantly increased in the 4-channel NMES group compared with that in the 2-channel NMES group (Table 4). In this study, one channel was attached to the suprahyoid muscles in the 2-channel NMES group, whereas the first and second channels were attached to the bilateral suprahyoid muscles in the 4-channel NMES group. Since the effective depth of NMES is related to the distance between the stimulation electrodes, a wider placement of the electrodes in the sequential 4-channel NMES might have been more effective in stimulating the suprahyoid muscles^26^.

The main shortcoming of the 4-channel NMES is that it is more cumbersome to attach electrodes compared to the 2-channel NMES. Additionally, the 4-channel NMES can have similar adverse events to conventional NMES, including discomfort, pain, burning sensation, and skin irritation^27^. In our study, one participant in the 2-channel NMES dropped out of the study due to discomfort (aggravated dizziness), which was not observed in the 4-channel NMES group; moreover, no complication related to the 4-channel NMES was noted. Despite the small sample size, our findings suggest that 4-channel NMES is a safe and well-tolerated treatment for dysphagia^13^.

This study has some limitations. First, data from only a small number of subjects of the original randomized controlled trial could be analyzed. This is probably because the clinical settings of VFSS were different between the rehabilitation centers, and in particular, the VFSS images from Jeju University Hospital were on a camcorder, so two-dimensional kinematic analysis could not be performed on this data. We also excluded some recordings from the Daegu-Patima General Hospital because recording was done at 10 frames/s. As a result, the number of VFSS images that could be kinematically analyzed was smaller in the control group containing more these hospitals. The present study was a multicenter prospective study, and the sample size could not be further increased. In the future, for an exact comparison according to the food form or fluid amount and more reliable results, it will be necessary to recruit sufficient patients calculated for the sample size. Second, the kinematic analysis procedure may have led to some measurement errors. The axes and variables can change with the patient's posture and neck movements which were not accounted for. However, a previous study stated that the correlation between automatic and manual analysis of swallowing was in the range of 0.972–0.996 (0.986 ± 0.010) for the X-axis and 0.939–0.998 (0.985 ± 0.018) for the Y-axis^28^. In our previous study on the AKAP^16^, the correlation between fully automatic and manual analyses of non-mandible-overlapped images was in the range of 0.932–0.997 (0.986 ± 0.017) for the X-axis and 0.975–0.999 (0.992 ± 0.006) for the Y-axis, which were equivalent or slightly higher correlations than those in other reports. Furthermore, in mandible-overlapped images, high correlations were also observed between fully automatic and manual analyses (0.959–0.997 for the X-axis and 0.974–0.998 for the Y-axis), which was not assessed in other reports. Third, we used pixels as the measure of distance, because some VF videos were free of a coin, which is utilized to adjust the size of the subject in the image during post-processing. So, in the current study, we used the comparable normalized parameters, and if the patient attaches a coin with known morphological characteristics to the chin during the videofluoroscopic swallowing study (VFSS), cm (mm) can be used as distance variables.

## Conclusion

Here, kinematic analysis parameters were used to quantitatively evaluate the mechanism by which treatment using the sequential 4-channel NMES brings about clinical improvement of swallowing. Compared with the 2-channel NMES, the sequential 4-channel NMES showed significant kinematic improvement of the hyolaryngeal complex. The sequential 4-channel NMES, through its activation of the suprahyoid, thyrohyoid, and other infrahyoid muscles at appropriate intervals, might lead to the physiologic circular movement of the hyoid bone, and therefore be an effective treatment for dysphagia. The disordered mechanism found in this study also provides a better understanding of NMES treatment for patients with dysphagia. However, further studies will be required to ascertain kinematic improvement of 4-channel NMES.

### Supplementary Information

Supplementary Figure S1.

Supplementary Figure S2.

Supplementary Legends.

## Data Availability

The datasets used and/or analysed during the current study available from the corresponding author on reasonable request.

## Abbreviations

NMES: Neuromuscular electrical stimulation
VFSS: Videofluoroscopic swallowing study
IDDSI: International dysphagia diet standardization initiative
PAS: Penetration-aspiration scale
VDS: Videofluoroscopic dysphagia scale
