# Supplementary figures and images for: Kinematic mechanism of the rehabilitative effect of 4-channel NMES: post-hoc analysis of a prospective randomized controlled study
Source: Sci Rep. 2023 Aug 18;13:13445. doi: 10.1038/s41598-023-40359-3 (PMC10439227; doi:10.1038/s41598-023-40359-3)

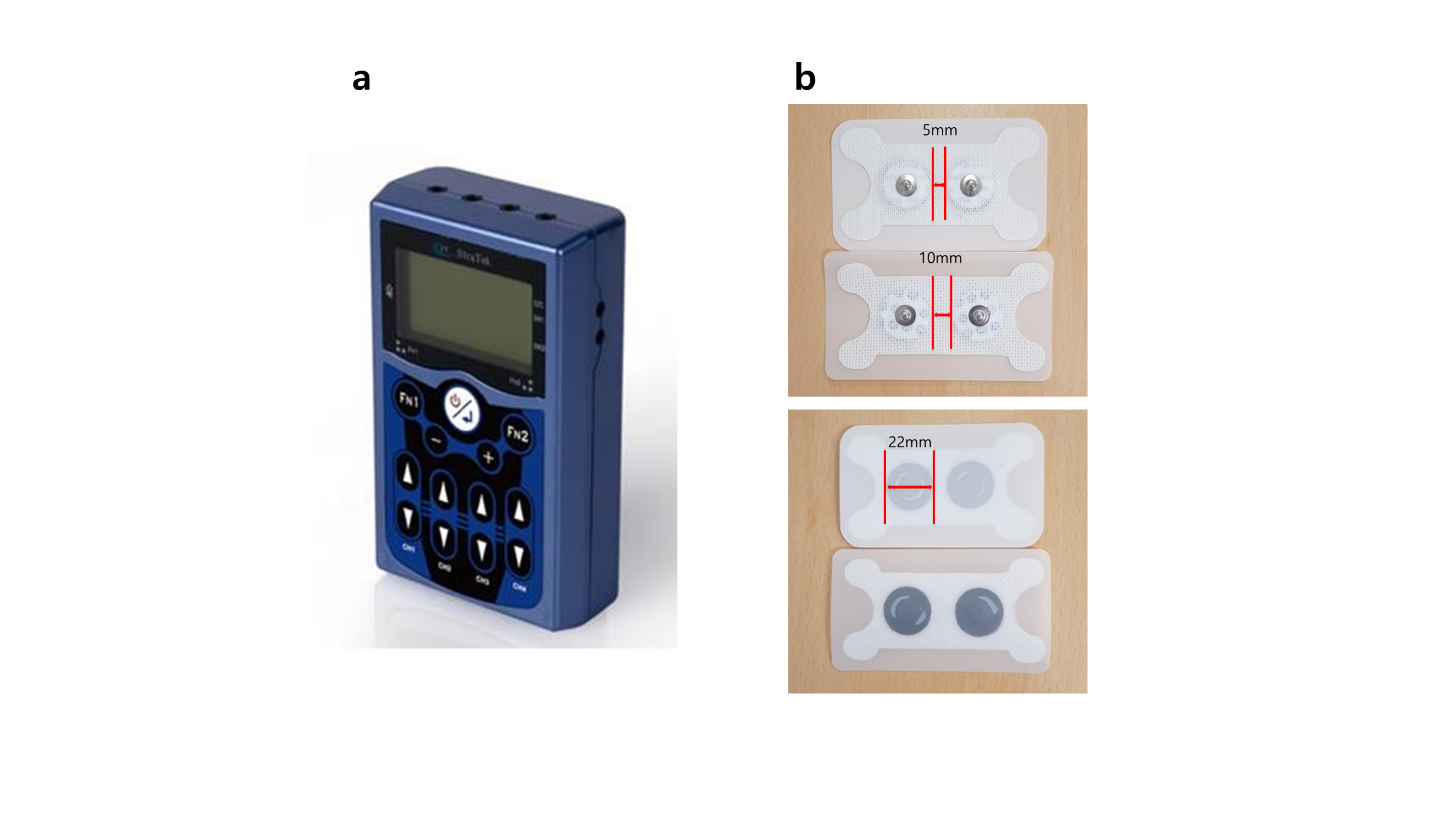

Supplement: Supplementary file 1 — Supplementary Figure S1. [file 41598_2023_40359_MOESM1_ESM.tif]

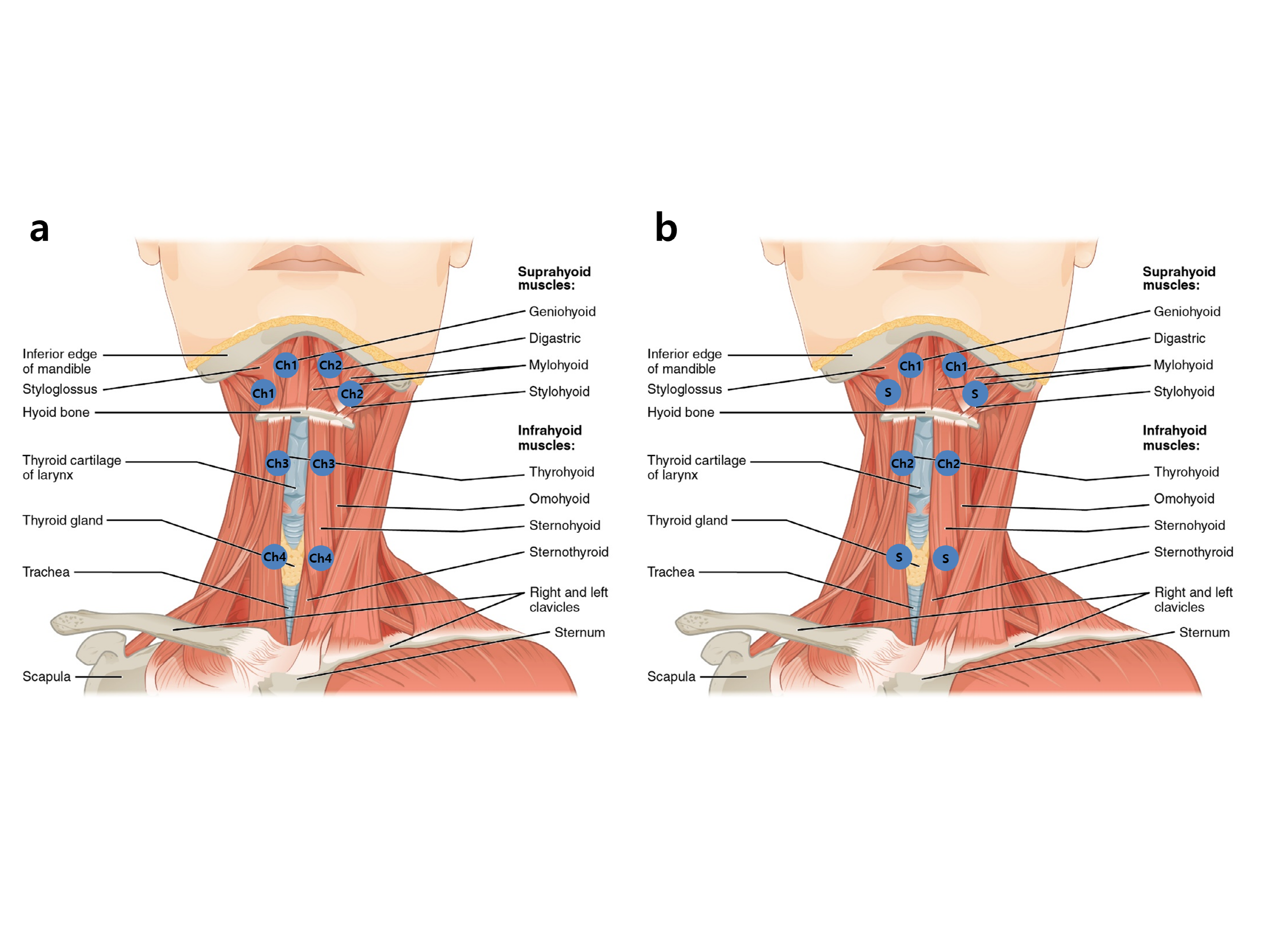

Supplement: Supplementary file 2 — Supplementary Figure S2. [file 41598_2023_40359_MOESM2_ESM.tif]
