# Supplementary material for: Kinematic mechanism of the rehabilitative effect of 4-channel NMES: post-hoc analysis of a prospective randomized controlled study
Source: Sci Rep. 2023 Aug 18;13:13445. doi: 10.1038/s41598-023-40359-3 (PMC10439227; doi:10.1038/s41598-023-40359-3)
Supplement: Supplementary file 3 — Supplementary Legends. [file 41598_2023_40359_MOESM3_ESM.docx]

**Kinematic mechanism of the rehabilitative effect of 4-channel NMES: post-hoc analysis of a prospective randomized controlled study**

Jiwoon Lim, MD^1^, Jun Chang Lee, PhD^1^, Eun Gyeong Jang, BS^1^, Sun Young Choi, BS^1^, Kyoung-Ho Seo, MD,PhD^2^, So Young Lee, MD^3^, Donghwi Park, MD^4^, Byung-Mo Oh, MD, PhD^5^, Han Gil Seo, MD, PhD^5^, Ju Seok Ryu, MD, PhD^1, 6^

^1^Department of Rehabilitation Medicine, Seoul National University Bundang Hospital, Seongnam, Korea

^2^Department of Rehabilitation Medicine, Seongnam Citizen’s Medical Center, Seongnam-si, South Korea

^3^Department of Rehabilitation Medicine, Jeju National University Hospital, Jeju National University College of Medicine, South Korea

^4^Department of Rehabilitation Medicine, Ulsan University Hospital, Ulsan, South Korea

^5^Department of Rehabilitation Medicine, Seoul National University College of Medicine, Seoul National University Hospital, South Korea

^6^Department of Rehabilitation Medicine, Seoul National University College of Medicine, Seoul, Korea

**Supplementary Figure S1.** The sequential 4-channel NMES device

**a** The device has four channels that are adjustable for amplitude of current, latency, and duration of electrical stimulation. The device uses four pairs of electrodes for electrical stimulation. **b** The electrodes are rounded and 22 mm long. The gaps between the electrodes are either 0.5 cm (type 1 electrode) or 1 cm (type 2 electrode). Type 1 electrode was used for channels 1, 2, and 4, and type 2 electrode was used for channel 3

**Supplementary Figure S2.** Locations of the electrode attachments

**a** Channel 1 (right) and channel 2 (left) electrodes were placed superior to the hyoid bone and posterior to the mandible 1 cm away from the midline, and the targeted muscles were the bilateral digastric and mylohyoid muscles. Channel 3 electrodes were placed on the bilateral superior pole of the thyroid cartilage to target the bilateral thyrohyoid muscles, and channel 4 electrodes were placed medial to the sternocleidomastoid muscles and inferior to the thyroid cartilage, and the targeted muscles were the other infrahyoid muscles (sternohyoid, omohyoid, and sternothyroid muscles). **b** In the 2-channel NMES system, channel 1 and 2 electrodes were attached to the suprahyoid and thyrohyoid muscles, respectively. Other electrodes were attached to the same locations as performed for the 4-channel NMES system, but stimulations were only applied to channel 1 and 2 electrodes. The copyright owner is OpenStax (Source: https://cnx.org/contents/FPtK1zmh@8.25:fEI3C8Ot@10/Preface). JW Lim recreated the drawing by adding electrodes to the original drawing)
